# Selective HDAC8 Inhibition Attenuates Isoproterenol-Induced Cardiac Hypertrophy and Fibrosis via p38 MAPK Pathway

**DOI:** 10.3389/fphar.2021.677757

**Published:** 2021-04-20

**Authors:** Tingwei Zhao, Hae Jin Kee, Liyan Bai, Moon-Ki Kim, Seung-Jung Kee, Myung Ho Jeong

**Affiliations:** ^1^ Heart Research Center of Chonnam National University Hospital, Gwangju, Republic of Korea; ^2^ Hypertension Heart Failure Research Center, Chonnam National University Hospital, Gwangju, Republic of Korea; ^3^ Department of Laboratory Medicine, Chonnam National University, Medical School and Hospital, Gwangju, Republic of Korea; ^4^ Department of Cardiology, Chonnam National University Medical School, Gwangju, Republic of Korea

**Keywords:** histone deacetylase8, PCI34051, cardiac hypertrophy, fibrosis, p38MAPK

## Abstract

Histone deacetylase (HDAC) expression and enzymatic activity are dysregulated in cardiovascular diseases. Among Class I HDACs, HDAC2 has been reported to play a key role in cardiac hypertrophy; however, the exact function of HDAC8 remains unknown. Here we investigated the role of HDAC8 in cardiac hypertrophy and fibrosis using the isoproterenol-induced cardiac hypertrophy model system.Isoproterenol-infused mice were injected with the HDAC8 selective inhibitor PCI34051 (30 mg kg^−1^ body weight). Enlarged hearts were assessed by HW/BW ratio, cross-sectional area, and echocardiography. RT-PCR, western blotting, histological analysis, and cell size measurements were performed. To elucidate the role of HDAC8 in cardiac hypertrophy, HDAC8 knockdown and HDAC8 overexpression were also used. Isoproterenol induced HDAC8 mRNA and protein expression in mice and H9c2 cells, while PCI34051 treatment decreased cardiac hypertrophy in isoproterenol-treated mice and H9c2 cells. PCI34051 treatment also reduced the expression of cardiac hypertrophic markers (Nppa, Nppb, and Myh7), transcription factors (Sp1, Gata4, and Gata6), and fibrosis markers (collagen type I, fibronectin, and Ctgf) in isoproterenol-treated mice. HDAC8 overexpression stimulated cardiac hypertrophy in cells, whereas HDAC8 knockdown reversed those effects. HDAC8 selective inhibitor and HDAC8 knockdown reduced the isoproterenol-induced activation of p38 MAPK, whereas HDAC8 overexpression promoted p38 MAPK phosphorylation. Furthermore, p38 MAPK inhibitor SB203580 significantly decreased the levels of p38 MAPK phosphorylation, as well as ANP and BNP protein expression, induced by HDAC8 overexpression.Here we show that inhibition of HDAC8 activity or expression suppresses cardiac hypertrophy and fibrosis. These findings suggest that HDAC8 could be a promising target to treat cardiac hypertrophy and fibrosis by regulating p38 MAPK.

## Introduction

Cardiac hypertrophy and fibrosis are common in hypertension, myocardial infarction, and heart failure (Kahan and Bergfeldt, 2005). Cardiac hypertrophy is characterized by the increase of heart mass, enhanced fetal gene expression, accelerated protein synthesis, and increased sarcomere structure, while fibrosis is described as the accumulation of extracellular matrix, such as collagen and fibronectin. Profibrotic factors secreted from the damaged tissues, activated immune cells, or various other cells, induce fibroblast differentiation into myofibroblasts (Duffield et al., 2013; Kendall and Feghali-Bostwick, 2014). Histone deacetylases (HDACs) are well known as the important regulators of gene expression in many biological processes (Haberland et al., 2009; Chen et al., 2015). HDAC inhibitors have shown beneficial effects on cardiac hypertrophy and fibrosis (Kee et al., 2006; Kang et al., 2015; Lee et al., 2016). These inhibitors improve myocardial function and prevent cardiac remodeling in diabetic mice (Chen et al., 2015). Pan-HDAC inhibitors or class I selective HDAC inhibitors also show an improvement in other various tissue type fibrosis-associated diseases, including liver and kidney (Van Beneden et al., 2013; Yoon et al., 2019).

HDACs have 18 different isoforms and are categorized into four subfamilies based on their sequence. Class I HDACs include HDAC1, HDAC2, HDAC3, and HDAC8. Among them, HDAC2 stimulates cardiac hypertrophy (Kee et al., 2008; Eom et al., 2011), whereas HDAC3 causes myocyte hyperplasia during embryonic and fetal life (Trivedi et al., 2008). HDAC2 selective inhibitor magnesium valproate has been reported to attenuate cardiac hypertrophy (Raghunathan et al., 2017). Furthermore, HDAC2 deficiency attenuated cardiac hypertrophy, whereas hypertrophy was augmented in HDAC2 transgenic mice (Trivedi et al., 2007). Among class I HDACs, the roles of HDAC1 and HDAC8 in cardiac hypertrophy remain unknown. HDAC8 is distributed both in the nucleus and cytoplasm and has been shown to regulate cortactin deacetylation (Li et al., 2014). In addition, HDAC2 and HDAC8 expression levels are upregulated in renovascular hypertensive rats, while sodium valproate, a non-specific HDAC inhibitor, attenuates cardiac remodeling, confirming the involvement of HDAC2 and HDAC8 in cardiac remodeling (Li et al., 2017). A recent study showed that HDAC8 expression is increased in lung tissues of patients with idiopathic pulmonary fibrosis, while HDAC8 inhibition ameliorates pulmonary fibrosis (Saito et al., 2019). In our previous studies, we demonstrated that the selective HDAC8 inhibitor, PCI34051, improved vascular hypertrophy in angiotensin II-induced hypertension (Kee et al., 2019) and the enzymatic activity of HDAC8 was increased in the hearts with deoxycorticosterone acetate-salt induced hypertension (Kee et al., 2013). PCI34051 also selectively inhibited the enzymatic activity of HDAC8 in a cell-free system (Kee et al., 2019).

We hypothesized that HDAC8 is associated with the development of cardiac hypertrophy and fibrosis. In this study, we investigated the role and regulatory mechanism of HDAC8 in isoproterenol-induced mouse and cellular models of cardiac hypertrophy. We showed that the inhibition of HDAC8 enzyme activity or expression attenuated cardiac hypertrophy, whereas HDAC8 overexpression increased cardiac hypertrophy. Our results suggest that HDAC8 is a novel target for the treatment of cardiac pathologies.

## Materials and Methods

### Reagents

PCI34051 (10,444) was purchased from Cayman Chemical Company (An Arbor, MI, United States), and isoproterenol (isoprenaline hydrochloride, I5627) and l-ascorbic acid (A7506) were purchased from Sigma-Aldrich Co. (St. Louis, MO, United States). Anti-GAPDH (sc-32233) and anti-BNP (sc-271185) antibodies were obtained from Santa Cruz Biotechnology (Dallas, TX, United States). Anti-p38 MAPK (8690) antibody, anti-phospho-p38 MAPK (4511) antibody, and SB203580 (5633) were purchased from Cell Signaling Technology (Danvers, MA, United States). Anti-ANP antibody was purchased from GeneTex (GTX109255; Irvine, CA, United States). Alexa Fluor 488 phalloidin was purchased from Invitrogen (Eugene, OR, United States). Anti-HDAC8 (17548-1-AP) antibody was purchased from Proteintech Group (Rosemont, IL, United States).

### Animal Model of Cardiac Hypertrophy

All animal procedures were approved by the Animal Experimental Committee of Chonnam National University Medical School (CNUH IACUC-18023) and were carried out according to the Guide for the Care and Use of Laboratory Animals (US National Institutes of Health Publications, eighth edition, 2011). The mice were maintained on a 12-h light/dark cycle under specific pathogen-free conditions.

Male CD-1 mice (7 weeks old and with an average weight of 33 g) were anesthetized with an intraperitoneal injection of ketamine (120 mg/kg) and xylazine (6.2 mg/kg). Isoproterenol was dissolved in 0.1% ascorbic acid and 0.9% saline, while PCI34051 was dissolved in dimethyl sulfoxide (DMSO) and diluted with 0.9% saline. Cardiac hypertrophy was induced by isoproterenol (25 mg/kg/day) infusion with an osmotic minipump (Alzet) for 5 days. The mice were randomly divided into three groups: vehicle control group (*n* = 8), isoproterenol-treated group (*n* = 8), and isoproterenol and PCI34051-treated group (*n* = 8; 30 mg/kg/ day) for 5 days.

### Echocardiography

Echocardiography was performed using a Vivid S5 echocardiography system (GE Healthcare, Chicago, IL, United States) with a 13-MHz linear array transducer. Mice were anesthetized with an intraperitoneal injection of tribromoethanol (Avertin; 114 mg/kg) before the procedure. M-mode (2-D guided) images and recordings were acquired from the long-axis view of the left ventricle at the level of the papillary muscles. The thickness of left ventricular posterior and interventricular septa was measured from the images.

### Histology and Picro-Sirius Red Staining

Mice were euthanized using a 100% CO_2_ for approximately 2–3 min. The hearts of mice were fixed with 3.7% paraformaldehyde and embedded in paraffin. The paraffin-embedded tissues were then cut into 4-µm sections, deparaffinized with xylene, and rehydrated in a series of graded alcohols. To measure cross-sectional cardiomyocyte area, tissue sections were stained with hematoxylin and eosin (H&E) as previously described (Ryu et al., 2016). Cross-sectional area was quantified using NIS Elements software (Nikon Eclipse 80i microscope, Tokyo, Japan). To determine cardiac fibrosis, Picro-Sirius Red staining (Abcam, Cambridge, United Kingdom) was performed. Sufficient Picro-Sirius Red solution was used to completely cover the rehydrated heart sections for 1 h. The slides were quickly rinsed two times with 0.5% acetic acid solution and then rinsed in absolute alcohol for 1 min. Next, the tissues were stained using the Gill’s hematoxylin for 4 min, rinsed with running tap water for 5 min, and dehydrated using 100% ethanol. After cleaning it in xylene, the slide was mounted using Canada balsam. Digital images were obtained with a microscope (Nikon, Japan) at 400x magnification.

### Reverse Transcription Polymerase Chain Reaction

Total RNA was isolated from heart tissues with TRIzol reagent (Invitrogen/Life Technologies, Carlsbad, CA, United States) and 1 μg was reverse transcribed with TOPscript RT DryMIX (Enzynomics, Daejeon, South Korea). mRNA levels were then quantified using a SYBR Green PCR kit (Enzynomics) using the 2^−∆∆Ct^ method. The PCR primers used in this study are listed in Table 1.

### Western Blotting

Total protein was extracted from heart tissues using RIPA lysis buffer (150 mM NaCl, 1% Triton X-100, 1% sodium deoxycholate, 50 mM Tris-HCl at pH 7.5, 2 mM EDTA, 1 mM PMSF, 1 mM DTT, 1 mM Na_3_VO_4_, and 5 mM NaF) containing a protease inhibitor cocktail (Calbiochem/EMD Millipore, Billerica, MA, United States). Proteins were separated using sodium dodecyl sulfate-polyacrylamide gel electrophoresis (SDS-PAGE), transferred to polyvinylidene difluoride membranes, and blocked with 5% skim milk in TBST buffer (20 mM Tris, 200 mM NaCl, and 0.04% Tween 20) for 1 h at 25°C. Next, the blots were incubated with primary antibodies overnight at 4°C, followed by anti-rabbit or anti-mouse horseradish peroxidase-conjugated secondary antibodies (diluted 1:5,000) for 1 h at 25°C. Protein bands were visualized using Immobilon western blotting detection reagents (EMD Millipore, Billerica, MA, United States). Bio-ID software (Vilber Lourmat, Eberhardzell, Germany) was used to quantify protein expression.

### Cell Culture and Cell Size Measurement

H9c2 cardiomyoblast cells were maintained in Dulbecco’s modified Eagle’s medium (DMEM) containing 10% fetal bovine serum (FBS) in 5% CO_2_ incubator at 37°C. For cell size experiments, cells were seeded on coverslips at a density of 1 × 10^4^/well, serum-starved overnight, and then treated with either vehicle or PCI34051 (100 nM) in the presence or absence of isoproterenol (10 μM) for the indicated time period. Cells were fixed with 3.7% paraformaldehyde, permeabilized with 0.1% Triton X-100, and incubated with Alexa Fluor 488 phalloidin (1:200) for 45 min, followed by 4′,6-diamidino-2-phenylindole (DAPI) staining. The cell size was measured using NIS Elements Software (Nikon, Japan).

### Cell Viability

H9c2 cells were seeded in 24-well plates and treated with different concentrations of PCI34051 (1, 10, 100 nM) or SB203580 (0.1, 1, 3, 10 μM) for 24 h. To measure viability, the cells were incubated with a 3-(4,5-dimethylthiazol-2-yl)-2,5-diphenyltetrazolium bromide (MTT) solution for 2 h, the insoluble formazan crystals were dissolved using DMSO, and the absorbance was measured at 570 nm.

### Transfection

To overexpress HDAC8, H9c2 cells were transfected with 1.6 μg of pCMV6-HA-myc or pCMV6-HDAC8-HA-myc plasmid for 2 days using Lipofectamine and PLUS reagents following the manufacturer’s protocol.

To knockdown HDAC8, H9c2 cells were transfected with control or HDAC8 siRNA (100 nM, Dharmacon, Lafayette, CO, United States) using RNAiMAX reagent according to the manufacturer’s instructions. The following day, the cells were serum-starved overnight and treated with isoproterenol for 9 h.

### Statistical Analysis

All data are expressed as mean ± standard error (SE). Statistical analysis was performed either by Student’s *t* test or one-way analysis of variance (ANOVA) and the Bonferroni post hoc test using GraphPad Prism version 5 (GraphPad Software, La Jolla, CA, United States). *p* values <0.05 were considered statistically significant.

## Results

### HDAC8 Selective Inhibitor Reduced Isoproterenol-Induced Cardiac Hypertrophy in Mice

**FIGURE 1:**
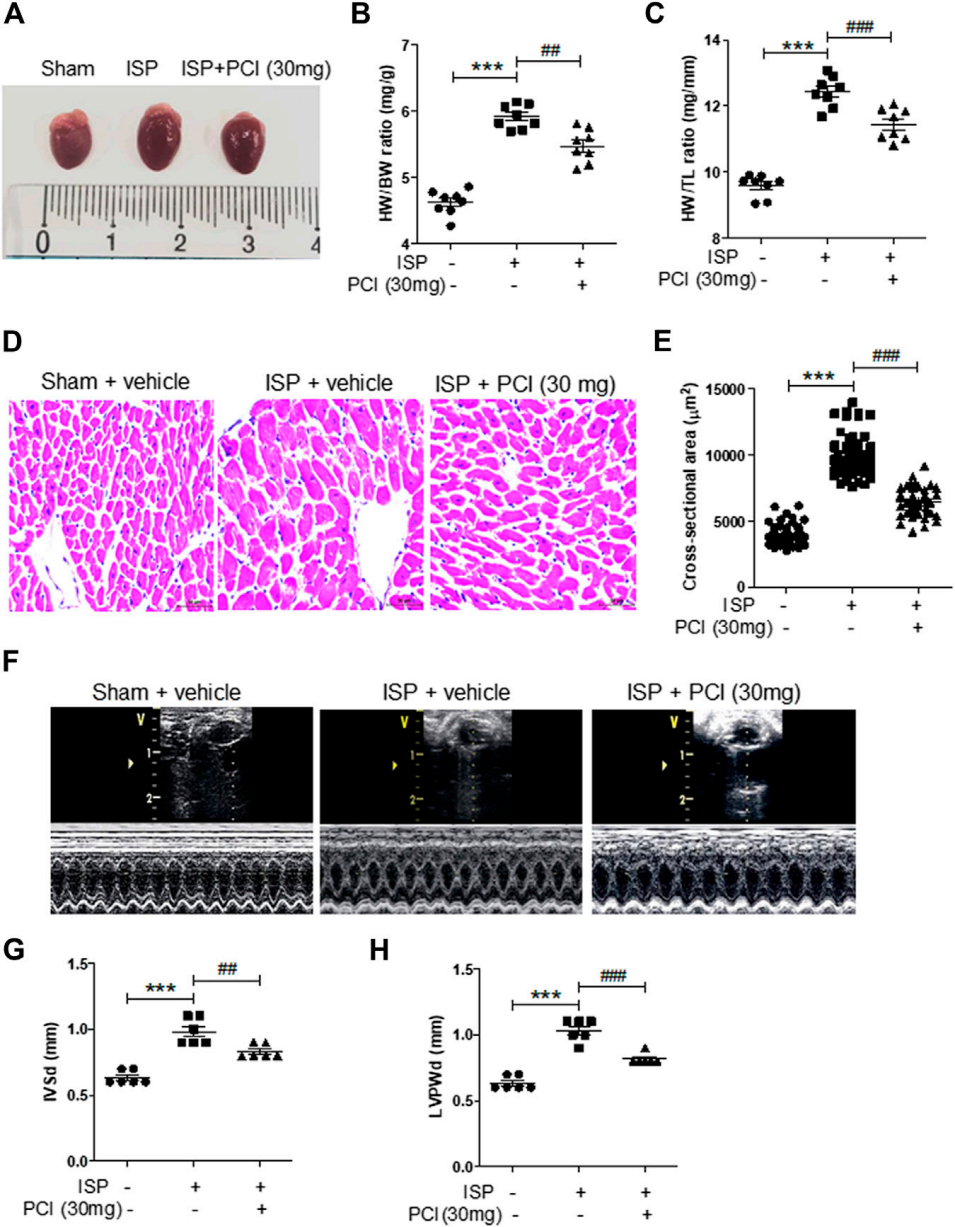
HDAC8 selective inhibitor reduces isoproterenol-induced cardiac hypertrophy in mice **(A)** Representative images of hearts from sham, isoproterenol (ISP), and ISP + PCI34051 (30 mg/kg/day) mice treated for 5 days **(B)** Heart weight to body weight (HW/BW) ratios (*n* = 8). ***p < 0.001; ##*p* < 0.01 **(C)** Heart weight to tibia length (HW/TL) ratios (*n* = 8). ***p < 0.001; ###*p* < 0.001 **(D)** H&E staining, representative images of heart tissues from sham, ISP, and ISP + PCI34051 (30 mg/kg/day) mice treated for 5 days (*n* = 8). Scale bar = 50 μm **(E)** Quantification of cardiomyocyte size cross-sectional area of samples described in **(D)**. ***p < 0.001; ###*p* < 0.001 **(F)** Representative M-mode echocardiograms after 5 days infusion of isoproterenol. PCI34051 was injected daily at 30 mg/kg/day for 5 days **(G–H)** Quantification of thickness of the left ventricular septum (IVSd) and posterior wall thickness (LVPWd) (*n* = 8). ***p < 0.001; ##*p* < 0.01 and ###*p* < 0.001. Data are presented as mean ± S.E. Statistics: one-way ANOVA followed by Bonferroni post hoc tests.

To determine whether HDAC8 is associated with the regulation of cardiac hypertrophy, we treated isoproterenol-infused mice with the selective HDAC8 inhibitor PCI34051 (30 mg/kg/day) for 5 days. As shown in Figures 1A–C, isoproterenol stimulation increased heart weight to body weight (HW/BW) ratio and heart weight to tibia length (HW/TL) ratio; however, this increase was reduced by PCI34051 treatment. The H&E staining was performed to analyze cardiac hypertrophy. Our results showed that the isoproterenol treatment increased cardiomyocyte size; however, the cell size was decreased by PCI34051 treatment (Figures 1D,E). To further investigate whether the HDAC8 selective inhibitor can affect cardiac wall thickness, we performed echocardiography. Isoproterenol infusion experiments increased the thickness of the interventricular septum and left ventricular posterior wall, while PCI34051 reduced those parameters (Figures 1F−H). Moreover, PCI34051 treatment significantly restored the isoproterenol-induced left ventricular internal dimension at end-systole; however, there were no significant differences between the left ventricular internal dimension results at end-diastole (Supplementary Figures S1A,B). Both ejection fraction and fractional shortening were increased by isoproterenol and then decreased in response to PCI34051 treatment (Supplementary Figures S1C,D).

### HDAC8 Selective Inhibitor Attenuated the Markers of Cardiac Hypertrophy and Fibrosis in Isoproterenol-Infused Mice

**FIGURE 2:**
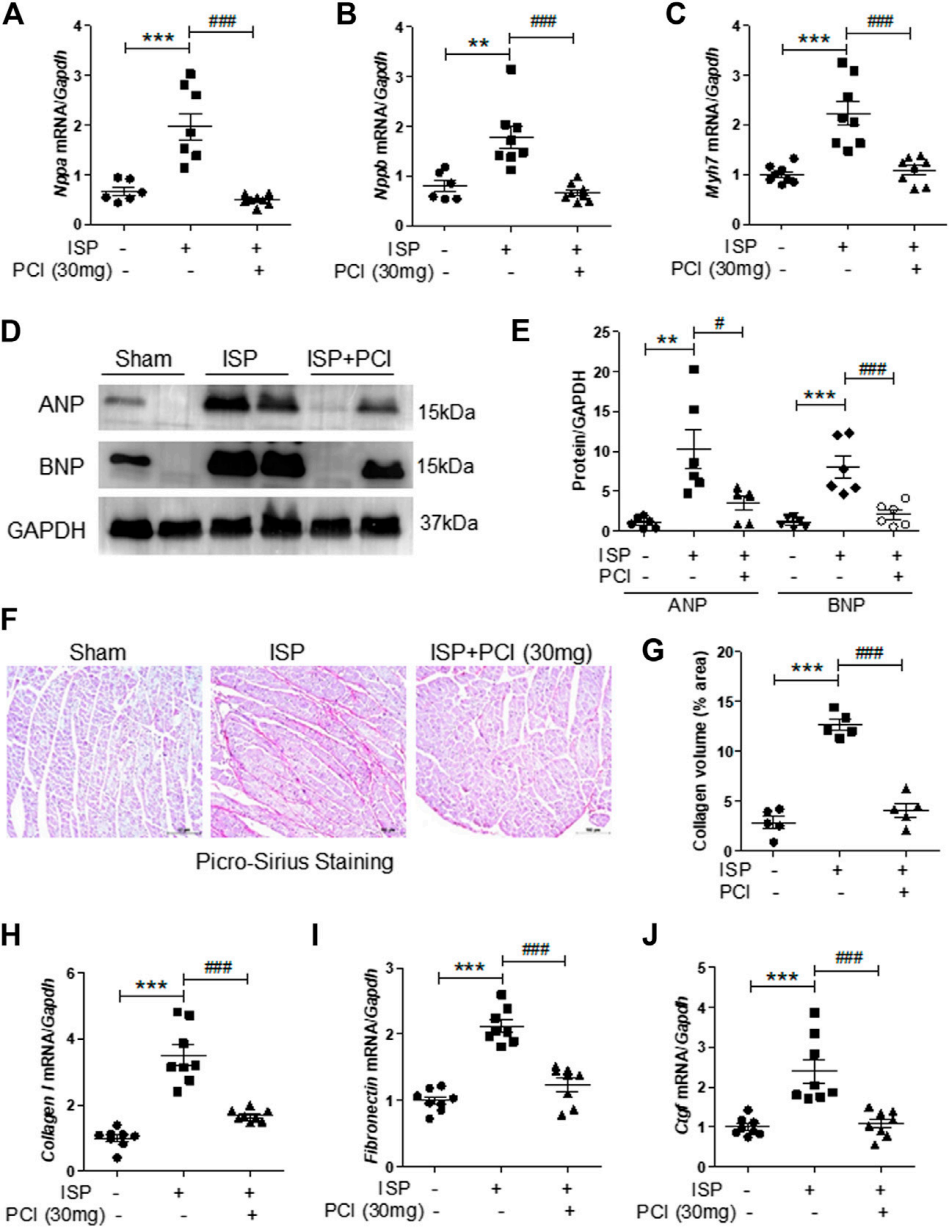
HDAC8 selective inhibitor attenuates cardiac hypertrophic marker genes and fibrosis in isoproterenol-infused mice.PCI34051 (30 mg/kg/day, PCI) was administered for 5 days after the infusion of isoproterenol **(A–C)** mRNA from heart tissues was evaluated by RT-PCR (*n* = 7–8) **(A)** Nppa **(B)** Nppb, and **(C)** Myh7. **p < 0.01; ***p < 0.001; ###*p* < 0.001 **(D)** Protein expression of ANP and BNP in heart tissues from sham, isoproterenol (ISP), and PCI34051 + ISP mice was analyzed by western blotting. GAPDH was used as a loading control. Representative blots are shown **(E)** Quantification of ANP and BNP protein levels (*n* = 6). **p < 0.01 and ***p < 0.001; #*p* < 0.05 and ###*p* < 0.001 **(F–G)** Representative images and quantification of Picro-Sirius Red staining of the heart tissues. Scale bar = 100 μm. Fibrosis markers, including collagen type I **(H)**, fibronectin **(I)**, and Ctgf **(J)**, were determined by RT-PCR. ***p < 0.001; ###*p* < 0.001. ISP and PCI indicate isoproterenol and PCI34051, respectively. Data are presented as mean ± S.E. Statistics: one-way ANOVA followed by Bonferroni post hoc tests.

We examined the effects of PCI34051 on expression of cardiac hypertrophic marker genes. Nppa, Nppb, and Myh7 mRNA levels were increased in the hearts of isoproterenol-infused mice and this upregulation was significantly reduced by the PCI34051 treatment (Figures 2A–C). Western blot analysis showed that ANP and BNP protein levels were higher in the hearts of isoproterenol-treated mice; however, this increase was reversed by the PCI34051 administration (Figures 2D,E). The mRNA levels of transcription factors, including Sp1, Gata4, and Gata6, were significantly decreased by the treatment with PCI34051 (Supplementary Figures S2A–C). To identify whether the PCI34051 treatment had an effect on cardiac fibrosis, the Picro-Sirius Red staining and RT-PCR were performed. The staining demonstrated that isoproterenol infusion increased collagen deposition (pink); however, this effect was significantly decreased by the PCI34051 treatment (Figures 2F,G). Collagen I, *fibronectin*, and Ctgf mRNA levels were significantly reduced by PCI34051 (Figures 2H−J), while smooth muscle α-actin (SMA) and TGF-β1 mRNA and protein levels were increased in isoproterenol-infused hearts compared to the sham group. This increase was also reversed by PCI34051 (Supplementary Figures S3A−D).

### HDAC8 Selective Inhibitor Blocked Isoproterenol-Induced Hypertrophy in H9c2 Cells

**FIGURE 3:**
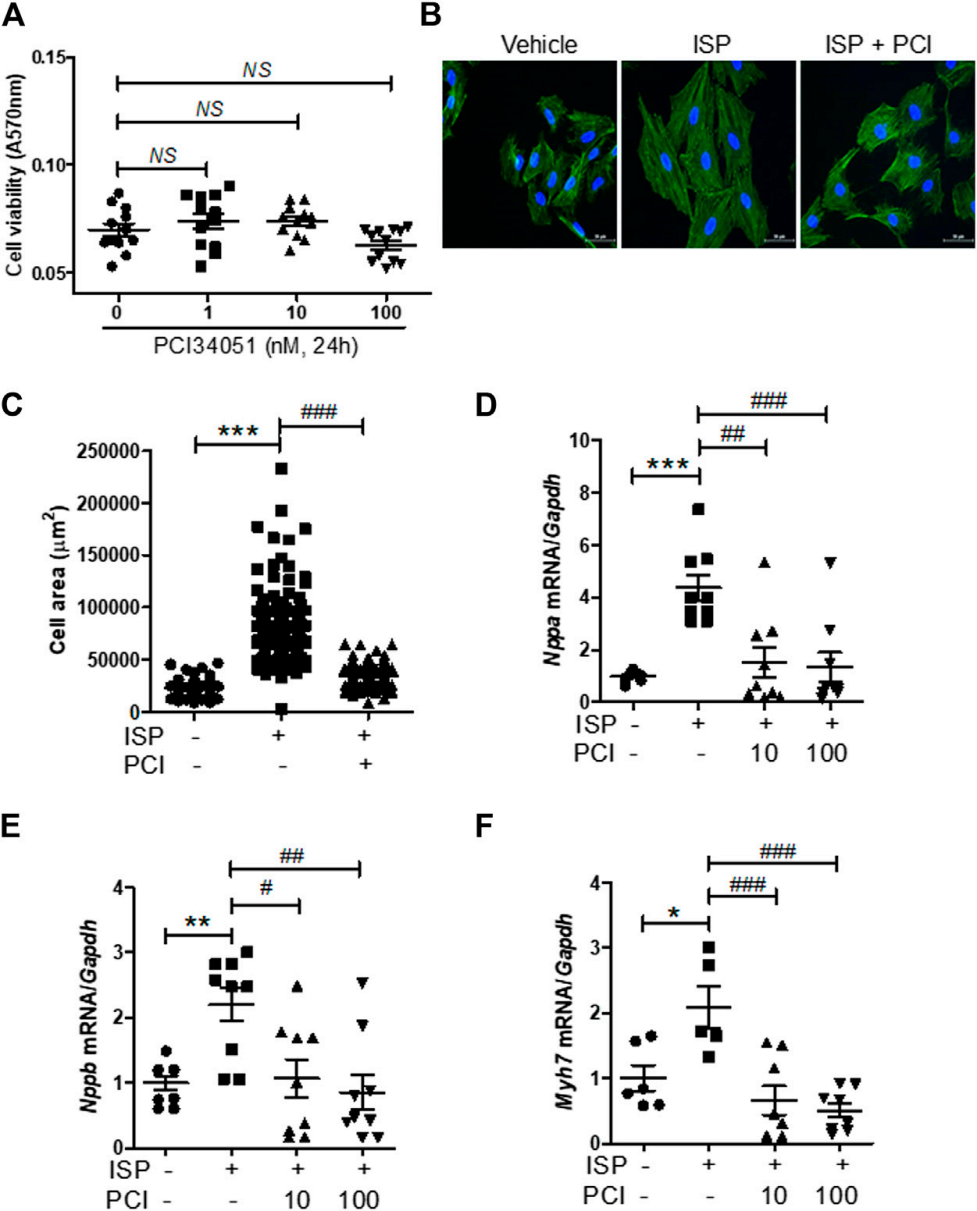
HDAC8 selective inhibitor attenuates isoproterenol-induced cardiac hypertrophy in H9c2 cells **(A)** H9c2 cells were treated with various concentrations of PCI34051 for 24 h and cell viability was evaluated (*n* = 12). NS = not significant **(B)** H9c2 cells were seeded in 12-well plates and serum starved overnight. Cells were treated with isoproterenol (10 μM) for 24 h in the presence of PCI34051 (100 nM) or vehicle (DMSO). Phalloidin staining was performed to determine cell size. Representative images **(B)** and quantification of cell size **(C)** are shown (*n* = 88–142). Scale bar = 50 μm ***p < 0.001; ###*p* < 0.001 **(D−F)** H9c2 cells were serum starved overnight and treated with vehicle or isoproterenol (10 μM, 6 h) in the presence or absence of PCI34051 (10 or 100 nM, 5 h). mRNA expression levels of Nppa **(D)**, Nppb **(E)**, Myh7 **(F)** were determined using RT-PCR (*n* = 6–9). *p < 0.05, **p < 0.01, and ***p < 0.001; #*p* < 0.05, ##*p* < 0.01, and ###*p* < 0.001. Data are presented as mean ± S.E. Statistics: one-way ANOVA followed by Bonferroni post hoc tests.

Next, we evaluated the cardiac protective effects of PCI34051 *in vitro* using the H9c2 cardiomyoblast cell line. First, we performed a dose response experiment. Cell viability was not affected at all tested concentrations (up to 100 nM) of PCI34051 (Figure 3A). The HDAC8 selective inhibitor PCI34051 effectively reduced the isoproterenol-induced increase of cardiomyocyte size (Figures 3B,C). To determine whether PCI34051 can affect cardiac hypertrophic marker genes, we performed RT-PCR. The isoproterenol treatment significantly increased the mRNA levels of Nppa, Nppb, and Myh7 in H9c2 cells (Figures 3D−F); however, 10 and 100 nM of PCI34051 reversed this effect. The expression of transcription factors Sp1, Gata4, and Gata6 was also increased in response to isoproterenol stimulation and this effect was decreased by PCI34051 (Supplementary Figures S4A−C).

### Overexpression of HDAC8 Induced Cardiac Hypertrophy in H9c2 Cells

**FIGURE 4:**
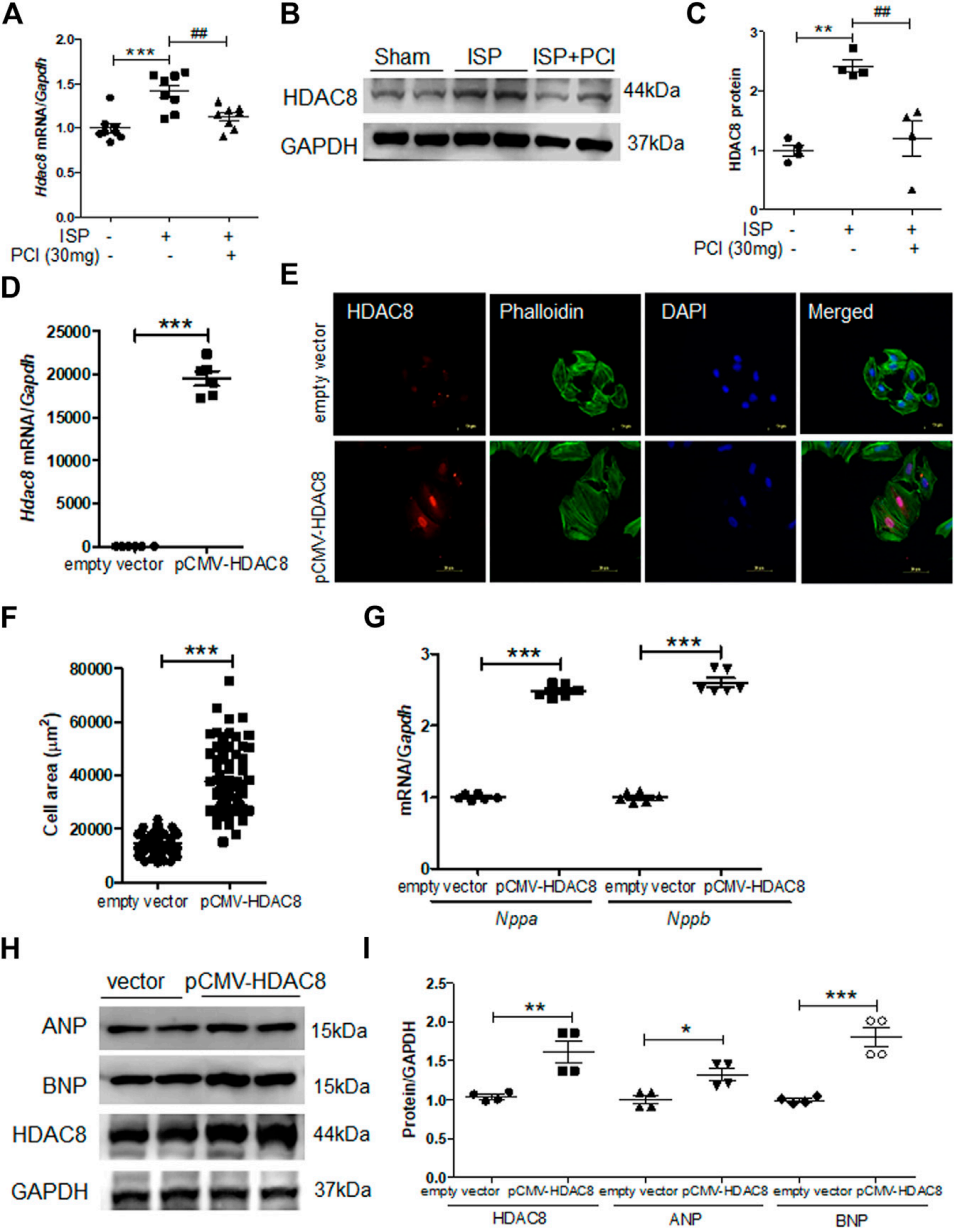
Overexpression of HDAC8 induces cardiac hypertrophy in H9c2 cells **(A)** mRNA expression levels of HDAC8 in heart tissues from sham, ISP, and ISP + PCI34051 (30 mg/kg/day)-treated mice (*n* = 8). ***p < 0.001; ##*p* < 0.01 from one-way ANOVA followed by Bonferroni post hoc tests **(B-C)** Western blot analysis of HDAC8 and GAPDH (loading control) from the samples described in **(A) (B)** Representative blots **(C)** Quantification of HDAC8 (*n* = 4). **p < 0.01; ##*p* < 0.01 from one-way ANOVA followed by Bonferroni post hoc tests **(D)** H9c2 cells were transfected with pCMV6-HA-Myc or pCMV6-HDAC8-HA-Myc for 48 h Hdac8 mRNA expression levels. ***p < 0.001 from the Student’s *t* test **(E)** The transfected cells were fixed and stained using anti-HDAC8 antibody and Alexa Fluor 488 phalloidin. Scale bar = 50 μm. Red, green, and blue indicate HDAC8, actin filaments, and nuclei, respectively **(F)** Quantification of cell size (*n* = 70–75). ***p < 0.001 from the Student’s *t* test **(G)** Nppa and Nppb mRNA expression levels in these cells (*n* = 6). ***p < 0.001 from the Student’s *t* test **(H)** Western blot analysis for ANP, BNP, and HDAC8. Representative blots **(I)** Quantification of HDAC8, ANP, and BNP protein levels (*n* = 4). *p < 0.05, **p < 0.01, and ***p < 0.001 from the Student’s *t* test.

To explore the role of HDAC8 in the isoproterenol-induced cardiac hypertrophy, the expression of HDAC8 was examined in hypertrophied mouse hearts. The mRNA levels of Hdac8 were significantly increased in isoproterenol-infused mouse hearts; however, this increase was reduced by the treatment with PCI34051 (Figure 4A). Similar results were obtained in the western blot analysis (Figures 4B,C). To test whether HDAC8 overexpression contributed to cardiac hypertrophy, H9c2 cells were transiently transfected with pCMV6-HDAC8-HA-myc plasmid. As expected, HDAC8 mRNA levels were higher in transfected cells compared with the control group (Figure 4D). HDAC8 overexpression significantly increased cell size (Figures 4E,F). Furthermore, HDAC8 overexpression increased the mRNA levels of Nppa and Nppb as confirmed by the RT-PCR analysis (Figure 4G). Consistent with gene expression results, HDAC8 overexpression resulted in higher protein levels of ANP and BNP compared with the control group (Figures 4H,I). We previously reported that HDAC2 directly induced cardiac hypertrophy and activated heat shock protein 70 (HSP70) in heart tissues (Kee et al., 2008). Therefore, we assessed whether there is a connection between HDAC8 and the expression of HDAC2 and HSP70. HDAC8 overexpression did not affect Hdac2 and Hsp70 mRNA levels (Supplementary Figures S5A,B).

### Knockdown of HDAC8 Reduced Isoproterenol-Induced Cardiomyocyte Hypertrophy in H9c2 Cells

**FIGURE 5:**
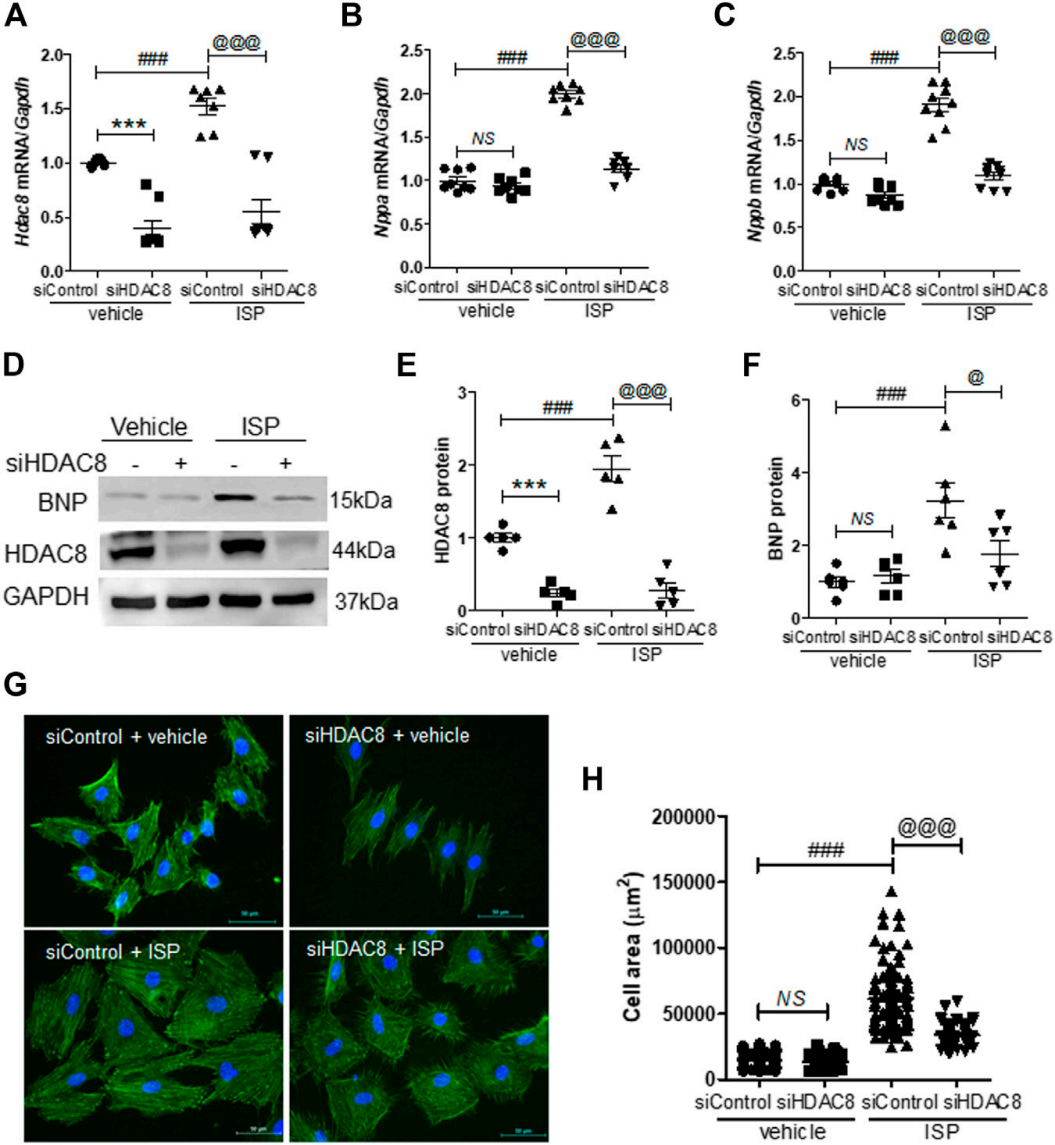
Knockdown of HDAC8 reduces isoproterenol-induced cardiomyocyte hypertrophy in H9c2 cells. H9c2 cells were transfected with control or HDAC8 siRNA and then stimulated with isoproterenol (10 μM) for 9 h **(A–C)** mRNA expression levels of Hdac8, Nppa, and Nppb in these cells (*n* = 8–9). ***p < 0.001; ###*p* < 0.001; ^@@@^ *p* < 0.001. NS = not significant **(D-F)** Representative western blot images **(D)** and quantification of HDAC8 **(E)** and BNP **(F)** protein levels (*n* = 5–6). ***p < 0.001; ###*p* < 0.001; ^@^ *p* < 0.05 and ^@@@^ *p* < 0.001 **(G)** Phalloidin-stained cells transfected with control or HDAC8 siRNA and then treated with isoproterenol **(H)** Quantification of cell size (*n* = 100–116). ###*p* < 0.001; ^@@@^ *p* < 0.001. NS = not significant. Data are presented as mean ± S.E. Statistics: one-way ANOVA followed by Bonferroni post hoc tests.

Next, we investigated the effect of HDAC8 downregulation on cardiac hypertrophy in H9c2 cells. For that purpose, we reduced the endogenous HDAC8 expression by transfecting H9c2 cells with HDAC8 siRNA. HDAC8 siRNA transfection successfully silenced the mRNA levels of Hdac8 (Figure 5A). Similar to our previous experiments, Hdac8 mRNA levels were significantly induced in response to the isoproterenol stimulation, and this response was reversed in the HDAC8 siRNA-transfected cells (Figure 5A). Furthermore, HDAC8 downregulation did not affect the mRNA levels of Nppa and Nppb; however, the isoproterenol-induced Nppa and Nppb mRNA expression was significantly reduced by transfection with HDAC8 siRNA (Figures 5B,C). Similar results were also obtained in the western blot analysis (Figures 5D−F). To test whether HDAC8 knockdown had an effect on the cardiomyocyte size, phalloidin staining was performed. As shown in Figures 5G,H, there was no significant difference in cell size between control and HDAC8 siRNA without hypertrophic stimuli; however, in response to the isoproterenol stimulation, the cell size was significantly decreased in HDAC8 siRNA-transfected cells compared to control cells.

### Inhibition or Silencing of HDAC8 Attenuated Isoproterenol-Induced p38 MAPK Signaling

**FIGURE 6:**
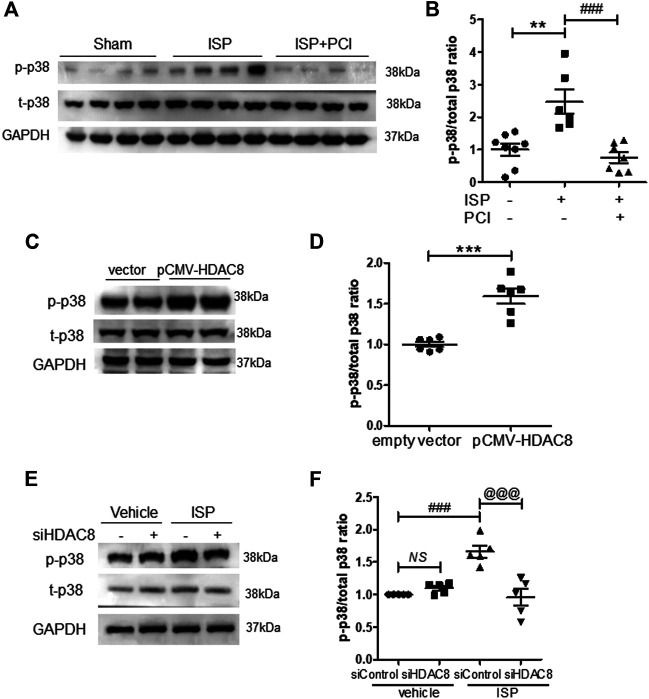
Inhibition or knockdown of HDAC8 attenuates isoproterenol-induced phosphorylation of p38 MAPK *in vivo* and *in vitro* **(A)** PCI34051 (30 mg/kg/day, PCI) was administered for 5 days after infusion of isoproterenol (ISP) in mice. Representative western blot images of phosphorylated p38 in heart tissues from sham, ISP, ISP + PCI34051 treated mice **(B)** Quantification of phosphorylated p38 protein levels (*n* = 6–8). **p < 0.01; ###*p* < 0.001 from one-way ANOVA followed by Bonferroni post hoc tests **(C)** H9c2 cells were transfected with pCMV6-HA-Myc or pCMV6-HDAC8-HA-Myc for 48 h. Representative western blot images of phosphorylated and total p38 in H9c2 cells **(D)** Quantification of phosphorylated p38 protein levels (*n* = 6). ***p < 0.001 from the Student’s *t* test **(E)** H9c2 cells were transfected with either control or HDAC8 siRNA and then stimulated with ISP for 9 h. Representative western blot images **(F)** Quantification of phosphorylated p38 (*n* = 5). ###*p* < 0.001; ^*@@@*^ *p* < 0.001 from one-way ANOVA followed by Bonferroni post hoc tests (**I**) Proposed model: HDAC8 functions as a novel therapeutic target in isoproterenol-induced cardiac hypertrophy.

To investigate whether HDAC8 regulates p38 MAPK signaling during the isoproterenol-induced cardiac hypertrophy, we performed western blot analysis. PCI34051 treatment decreased the phosphorylation levels of p38 in isoproterenol-stimulated hearts (Figures 6A,B). Next, we evaluated the effect of HDAC8 overexpression on p38 MAPK protein expression and activity. HDAC8 overexpression did not change total p38 MAPK protein levels; however, p38MAPK phosphorylation was increased (Figures 6C,D). Next, we assessed p38 MAPK expression in HDAC8 siRNA-transfected H9c2 cells. The knockdown of HDAC8 also significantly reduced the levels of phosphorylated p38 MAPK protein induced by isoproterenol (Figures 6E,F).

### Inhibition of p38 MAPK Reduces the Expression of Hypertrophic Markers

**FIGURE 7:**
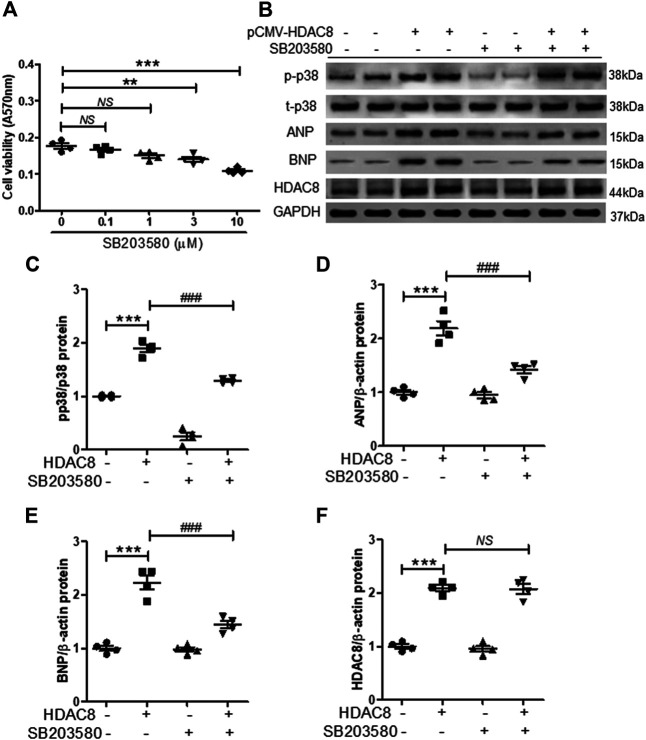
Inhibition of p38 MAPK reduces the expression of cardiac hypertrophic markers induced by HDAC8 overexpression **(A)** H9c2 cells were treated with SB203580 (0.1, 1, 3, and 10 μM) for 24 h and cell viability was evaluated (*n* = 4). **p < 0.01 and ***p < 0.001. NS = not significant **(B)** H9c2 cells were transfected with either pCMV6-HA-Myc or pCMV6-HDAC8-HA-Myc and treated with SB203580 (1 μM) for 24 h. Representative western blot images of p-p38, t-p38, ANP, BNP, and HDAC8 **(C−F)** Quantification of p-p38, ANP, BNP, and HDAC8 protein levels (*n* = 4). ***p < 0.001; ###*p* < 0.001. NS = not significant.

To further investigate the association between HDAC8 and p38 MAPK in cardiac hypertrophy, H9c2 cells were transfected with the pCMV6-HDAC8-HA-Myc plasmid and treated with p38 MAPK inhibitor SB203058 (1 μM; this concentration of SB203058 did not affect cell viability (Figure 7A). As expected, HDAC8 overexpression increased the levels of phosphorylated p38 MAPK, ANP, and BNP (Figures 7B−E), whereas SB203058 treatment significantly reduced the levels of phosphorylated p38 MAPK (Figures 7B,C). Interestingly, SB203058 treatment also decreased protein levels of ANP and BNP (Figures 7B−E); however, HDAC8 protein expression was not affected by p38 MAPK inhibitor treatment (Figures 7B,F).

## Discussion

**FIGURE 8:**
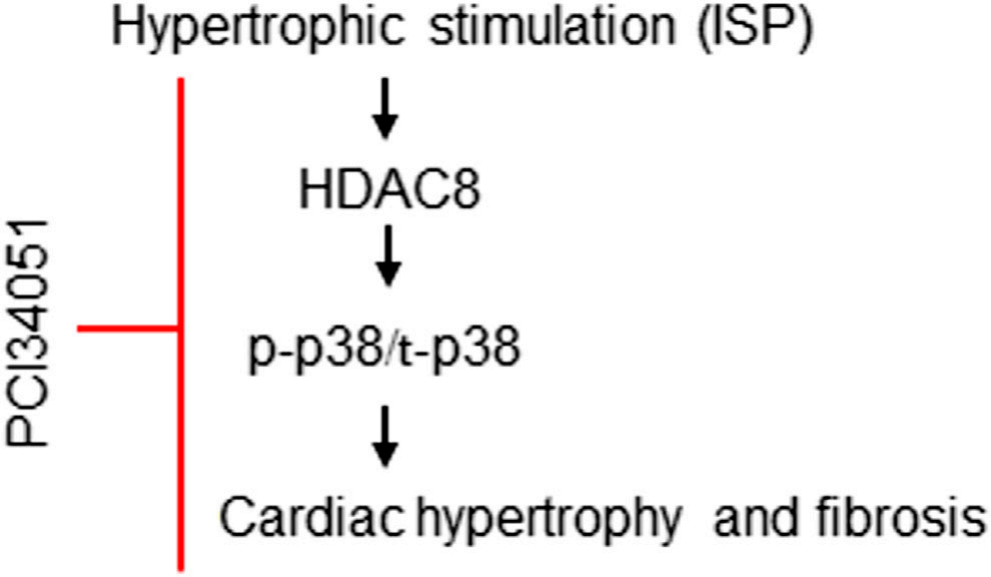
Proposed role of HDAC8 in cardiac hypertrophy and fibrosis. Beta-adrenergic agonist isoproterenol increases the expression of HDAC8. Higher levels of HDAC8 induce p38 MAPK phosphorylation and increase the expression of cardiac hypertrophic markers ANP and BNP. PCI34051, a selective inhibitor of HDAC8, retards cardiac hypertrophy and fibrosis by inhibiting p38 MAPK activity.

In this study, we identified HDAC8 as a mediator of hypertrophy and fibrosis. Our results showed that isoproterenol, a non-selective beta adrenergic agonist, induced HDAC8, cardiac hypertrophic marker genes, p38 MAPK, and fibrosis-related genes. The treatment with PCI34051, an HDAC8 selective inhibitor, ameliorated cardiac hypertrophy and fibrosis (Figure 8). Furthermore, we observed that cardiac hypertrophy was successfully reversed by PCI34051. Our findings also demonstrated that PCI34051 was well tolerated in mice at the concentrations of up to 30 mg/kg/day.

Among the 18 mammalian HDACs, HDAC2 acts as a pro-hypertrophic regulator of cardiac hypertrophy development by modulating the GSK3β or kruppёl like factor 4 activity (Trivedi et al., 2007; Kee et al., 2008; Kee and Kook, 2009). HSP70 is the interacting partner of HDAC2 and its expression is increased by hypertrophic stimuli. Indeed, Hsp70 mRNA levels were increased in response to the isoproterenol stimulation (Supplementary Figure S7). In this study, HSP70 did not physically interact with HDAC8 (Supplementary Figure S7), suggesting that the association with HSP70 was specific to HDAC2. Moreover, HDAC8 did not influence Hdac2 and Hsp70 expression. These findings indicate that HDAC8 has a distinct regulatory mechanism in the cardiac hypertrophy control.

Here we present novel findings demonstrating that, besides HDAC2, HDAC8 is directly implicated in cardiac hypertrophy. We elucidated the role of HDAC8, first, by inhibiting HDAC8 activity (using a specific pharmacological inhibitor and siRNA), and, second, by overexpressing HDAC8. PCI34051, an HDAC8 selective inhibitor, attenuated cardiac hypertrophy, as determined by HW/BW and HW/TL ratios, cross-sectional areas, and left ventricular posterior and septum thickness in isoproterenol-infused mice. In the H9c2 cells, the isoproterenol-induced increase in cell size was reduced by the PCI34051 treatment. PCI34051 also significantly suppressed the expression of cardiac hypertrophic marker genes, including Nppa, Nppb, Myh7, as well as transcription factors Sp1, Gata4, and Gata6, both *in vivo* and *in vitro*. These observations demonstrate that HDAC8 could be a novel therapeutic target for the treatment of cardiac hypertrophy.

To confirm the involvement of HDAC8 in the regulation of cardiac hypertrophy, we used a selective inhibitor and a knockdown technique. Previous studies reported that non-specific class I HDAC or pan-HDAC inhibitors suppressed cardiac hypertrophy (Cao et al., 2011; Morales et al., 2016). In the present study, PCI34051, the selective inhibitor of HDAC8 (a class I HDAC) was used to demonstrate the pro-hypertrophic function of HDAC8. Our previous study showed that the treatment of vascular smooth muscle cells with PCI34051 attenuated the angiotensin II-induced vascular hypertrophy through the reduction of GATA6 expression (Kee et al., 2019). Here we observed that the HDAC8 selective inhibitor significantly reduced Gata4 and Gata6 mRNA levels in H9c2 cells.

One of the interesting findings was the observation that HDAC8 mRNA and protein levels increased in response to the isoproterenol stimulation. In addition to the hypertrophic stimulus, HDAC8 can be induced by the exposure to other stresses, such as hypoxia and UV irradiation (Emmons et al., 2019). Unlike HDAC8, the enzyme activity of another class I HDAC, HDAC2, but not the expression, was increased in response to several hypertrophic stimuli (Kee et al., 2008). Furthermore, the phosphorylation of HDAC2 by casein kinase 2α1 is required for cardiac hypertrophy (Eom et al., 2011). However, the significance of HDAC8 phosphorylation in cardiac hypertrophy is not yet known. Overexpression of HDAC2 in cardiomyocytes or in transgenic mice induces hypertrophy (Trivedi et al., 2007; Kee et al., 2008). In the present study, the overexpression of HDAC8 in H9c2 cells resulted in an increased cell size and higher levels of hypertrophic marker genes. These findings suggest that HDAC8 is directly related to the induction of cardiac hypertrophy.

Several HDAC inhibitors have been demonstrated to inhibit fibrosis following a variety of stimuli (Nural-Guvener et al., 2014; Yoon et al., 2019). Although there is no direct evidence of the HDAC2 involvement in fibrogenesis, it was reported that HDAC2 knockdown decreased collagen type I and SMA in cardiac fibroblasts (Jin et al., 2017; Yang et al., 2020). We showed that HDAC8 regulated cardiac fibrosis-related genes *in vivo*. The PCI34051 treatment reduced cardiac fibrosis in heart tissues. The fibrotic markers, including collagen type I, fibronectin, Ctgf, SMA, and TGF-β1, were significantly decreased by the HDAC8 selective inhibitor. The Picro-Sirius Red staining showed reduced collagen deposition in heart tissues treated with the HDAC8 selective inhibitor. Based on our results, we propose that HDAC8 induces cardiac fibrosis.

Our results showed that HDAC8 overexpression enhanced the mRNA and protein levels of hypertrophic markers (ANP and BNP). In addition to heart, HDAC8 has been reported to play an important role in the brain skull development (Haberland et al., 2009). In humans, loss-of-function HDAC8 mutations cause Cornelia de Lange syndrome with multisystem genetic disorders (Kaiser et al., 2014). HDAC8 is also associated with cancer (Chakrabarti et al., 2015). We and another group showed that HDAC8 is implicated in vascular contractility in hypertension (Li et al., 2014; Kee et al., 2019).

Many signaling pathways, including the MAPK pathway, calcineurin, and small G proteins, are involved in promoting cardiac hypertrophy (Molkentin et al., 1998; Ruwhof and van der Laarse, 2000; Frey et al., 2004). Here we show that p38 MAPK activation was observed in response to the isoproterenol stimulation both in heart tissues and in cells. Furthermore, p38 MAPK phosphorylation was suppressed by HDAC8 inhibition or knockdown and increased by HDAC8 overexpression. These observations suggest that p38 MAPK is a downstream target of HDAC8 and is implicated in the development of cardiac hypertrophy. There are conflicting reports regarding the role of p38α in the regulation of cardiac hypertrophy. For example, cardiac-specific p38α knockout mice did not show cardiac hypertrophy compared to control mice (Nishida et al., 2004), whereas fibroblast-specific p38α knockout mice were protected from isoproterenol-induced cardiac hypertrophy and fibrosis (Bageghni et al., 2018). These differences are possibly due to a cell-specific response (cardiomyocytes vs. fibroblasts). Furthermore, p38α was shown to be related to pressure-overload-induced heart failure, while p38β was found to be associated with compensatory hypertrophy (Hunter and Chien, 1999).

In response to various injuries or stress, cardiac fibroblasts contribute to the processes involved in cardiac remodeling, including inflammation, fibrosis, and hypertrophy. p38 MAPK plays a central role in regulating fibrosis (Turner and Blythe, 2019). Consistent with our findings, a previous study showed that the activation of p38 MAPK contributes to right ventricular hypertrophy and fibrosis (Kojonazarov et al., 2017). The authors of the study demonstrated that either p38 inhibitor or p38 knockdown suppressed TGF-β-induced SMAD2/3 phosphorylation and SMA expression. In addition, p38 inhibitor (SB203580) reportedly reduced pressure overload-induced left ventricular cardiac hypertrophy and fibrosis (Zhang et al., 2019). Consistent with these findings, here we showed that p38 MAPK inhibitor SB203058 decreased the expression of cardiac hypertrophy markers ANP and BNP, while their levels were increased by HDAC8 overexpression. However, in addition to p38 MAPK inhibition, SB203580 also affects the function of other kinases: it has been reported that this inhibitor decreased the phosphorylation of cAMP response element-binding protein by casein kinase 1 (Shanware et al., 2009).

microRNAs are another class of molecules involved in the regulation of gene expression. Yan et al. reported that miR-21–3p regulates cardiac hypertrophy by targeting HDAC8. The authors also showed that HDAC8 regulates cardiac hypertrophy via the Akt/GSK3β pathway (Yan et al., 2015), further supporting our findings.

One of the limitations of our study was the use of a single hypertrophic stimulation, both *in vivo* and *in vitro*. Additional pathological stresses, such as pressure-overload or hypertension, could have been used in the animal model to further confirm our results. Another limitation is the use of H9c2 rat cardiomyoblast cell line as the *in vitro* model system. Even though H9c2 cells respond to cardiac hypertrophy in a manner very similar to primary cardiomyocytes, H9c2 cells lack mature sarcomere organization and do not spontaneously beat (Peter et al., 2016).

In conclusion, our results demonstrated that HDAC8 functions as a pro-hypertrophic mediator in the heart. We provide evidence that HDAC8 activity and expression partially contribute to the development of cardiac hypertrophy. Targeting HDAC8 could be a novel therapeutic strategy for the treatment of cardiac hypertrophy.

## Data Availability

The original contributions presented in the study are included in the article/Supplementary Material, further inquiries can be directed to the corresponding authors.
