# Supplementary material for: Selective HDAC8 Inhibition Attenuates Isoproterenol-Induced Cardiac Hypertrophy and Fibrosis via p38 MAPK Pathway
Source: Front Pharmacol. 2021 Apr 20;12:677757. doi: 10.3389/fphar.2021.677757 (PMC8093872; doi:10.3389/fphar.2021.677757)
Supplement: Supplementary file 1 [file datasheet1.pdf]

Supplementary Figure Legends

Online Figure 1. PCI34051 treatment restores echocardiographic parameters in the isoproterenol-infused cardiac hypertrophy mouse model.

(**A** and **B**) Left ventricular internal diameters (LVIDs and LVIDd) at end-systole or end-diastole in isoproterenol-infused mice with or without PCI34051 treatment (30 mg/kg/day for 5 days, n=6). $^{***}P<0.001$, $^{\#\#}P<0.01$. NS = not significant. (**C** and **D**) Ejection fraction (EF, %) and fractional shortening (FS, %) in mice described in (**A-B**). $^{***}P<0.001$, $^{\#\#}P<0.01$, and $^{\#\#\#}P<0.001$. Data are presented as the mean ± S.E. Statistics: one-way ANOVA followed by Bonferroni *post hoc* tests.

Online Figure 2. The HDAC8 selective inhibitor attenuates the expression of cardiac-specific transcription factors in isoproterenol-infused mice.

PCI34051 (30 mg/kg/day) was administered for 5 days after infusing mice with isoproterenol. The mRNA expression levels of *Sp1* (**A**), *Gata4* (**B**), and *Gata6* (**C**) were evaluated by RT-PCR. $^{***}P<0.001$; $^{\#\#\#}P<0.001$. Data are presented as mean ± S.E. Statistics: one-way ANOVA followed by Bonferroni *post hoc* tests.

Online Figure 3. HDAC8 selective inhibitor attenuates the expression of cardiac fibrosis genes in isoproterenol-infused mice.

PCI34051 (30 mg/kg/day) was administered for 5 days after infusing mice with isoproterenol. The mRNA expression levels of *Sma* (**A**) and *Tgfb1* (**B**) were determined by RT-PCR (n=8). $^{***}P<0.001$; $^{\#\#\#}P<0.001$. (**C**) Protein levels of SMA and TGF-β1 were determined by western blotting. Representative images of the blots are shown. (**D**) Quantification of SMA and TGF-β1 protein levels (n=6). $^{*}P<0.05$ and $^{***}P<0.001$; $^{\#}P<0.05$ and $^{\#\#}P<0.01$. Data are presented as mean ± S.E. Statistics: one-way ANOVA followed by Bonferroni *post hoc* tests.

Online Figure 4. HDAC8 selective inhibitor reduces the mRNA expression of transcription factors in H9c2 cells stimulated with isoproterenol.

H9c2 cells were treated with vehicle or isoproterenol (10 μM, 6 h) in the presence or absence of PCI34051 (10 or 100 nM, 5 h). mRNA expression levels of *Sp1* (**A**), *Gata4* (**B**), and *Gata6* (**C**) were determined using RT-PCR (n=6–9). $^{**}P<0.01$; $^{\#}P<0.05$ and $^{\#\#}P<0.01$. Data are presented as mean ± S.E. Statistics: one-way ANOVA followed by Bonferroni *post hoc* tests.

Online Figure 5. HDAC8 overexpression does not affect *Hdac2* and *Hsp70* mRNA levels

in H9c2 cells.

H9c2 cells were transfected with *pCMV6-HA-Myc* or *pCMV6-Hdac8-HA-Myc* for 48 h. The mRNA levels of *Hdac2* (**A**) and *Hsp70* (**B**) were determined using RT-PCR (n=10–12). NS indicates not significant (Student's *t* test).

Online Figure 6. HDAC8 selective inhibitor or HDAC8 knockdown does not affect isoproterenol-induced *Hsp70* mRNA levels *in vivo* and *in vitro*.

(**A**) PCI34051 (30 mg/kg/day) was administered for 5 days after infusing mice with isoproterenol. The mRNA expression levels of *Hsp70* were determined by RT-PCR (n=8). ***$P<0.001$. NS = not significant. (**B**) H9c2 cells were transfected with control or HDAC8 siRNA and stimulated with isoproterenol for 9 h. *Hsp70* mRNA expression levels were determined by RT-PCR (n=6–8). $^{###}P<0.001$. NS = not significant. Data are presented as mean ± S.E. Statistics: one-way ANOVA followed by Bonferroni *post hoc* tests.

Online Figure 7. HDAC8 does not interact with HSP70 in H9c2 cells.

Immunoprecipitation using the anti-HDAC8 antibody was performed as previously described [1]. Endogenous HDAC8 was enriched with anti-HDAC8 antibody in H9c2 cells. Western blotting was performed with anti-HSP70, anti-p38, and anti-p-p38 antibodies.

References

[1] G.R. Kim, S.N. Cho, H.S. Kim, S.Y. Yu, S.Y. Choi, Y. Ryu, M.Q. Lin, L. Jin, H.J. Kee, M.H. Jeong, Histone deacetylase and GATA-binding factor 6 regulate arterial remodeling in angiotensin II-induced hypertension, J Hypertens 34(11) (2016) 2206-19.

Supplementary Figure 1

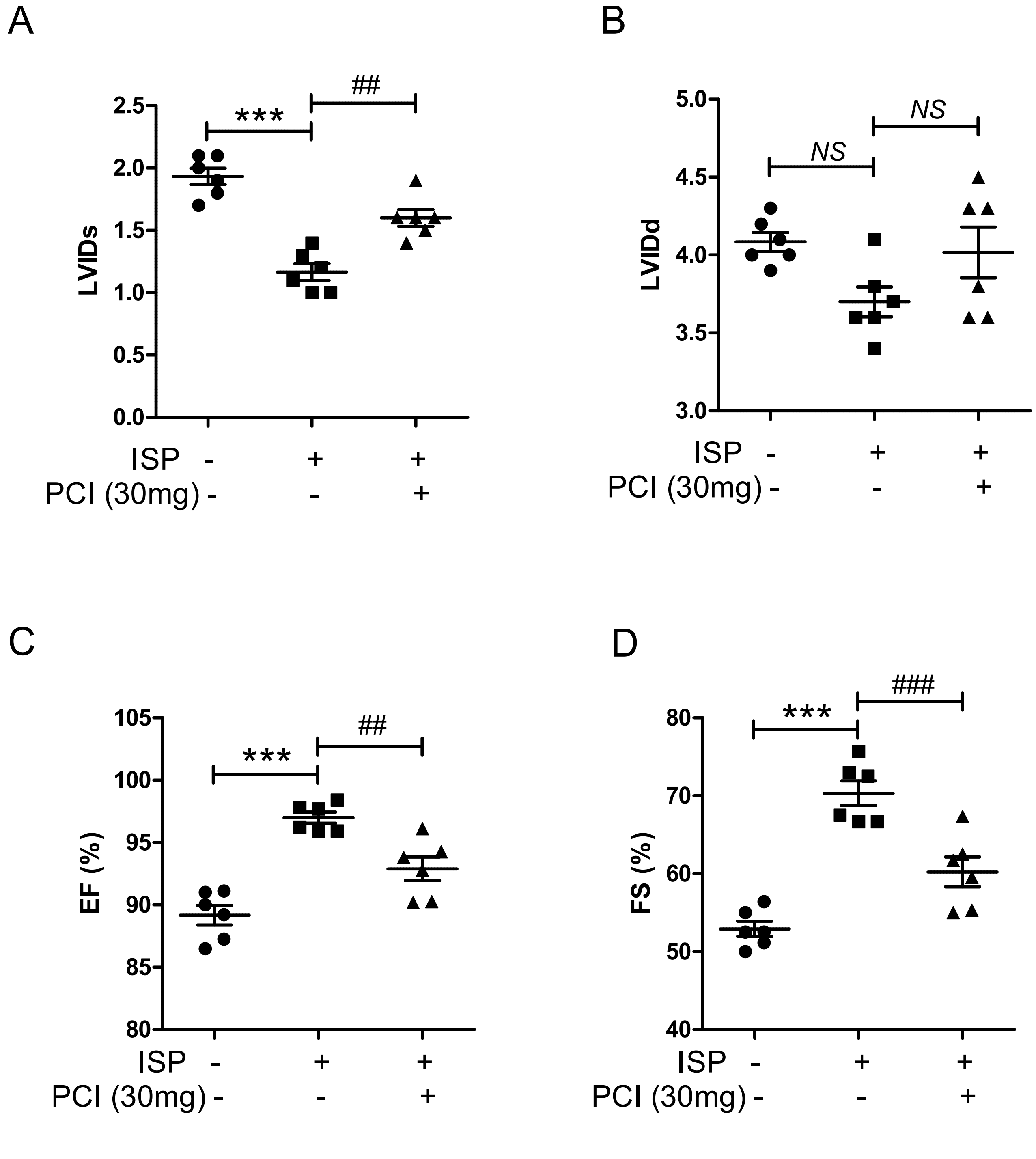

Supplementary Figure 2

A

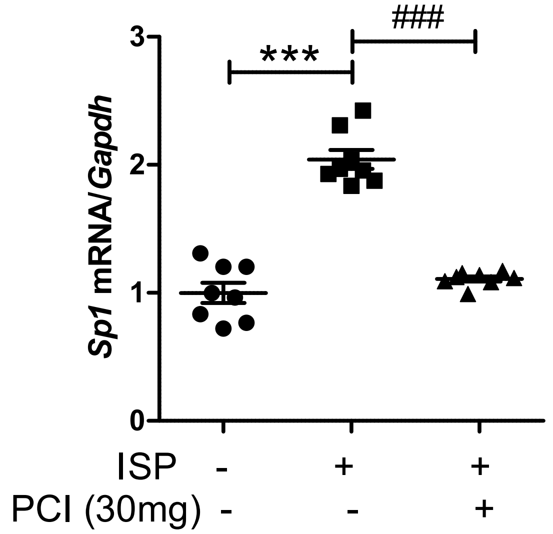

B

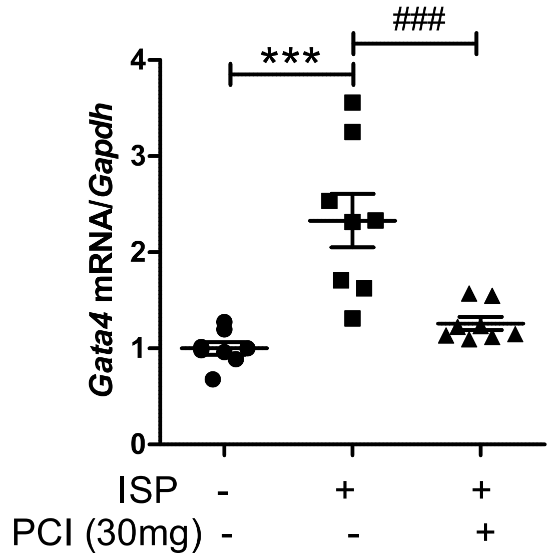

C

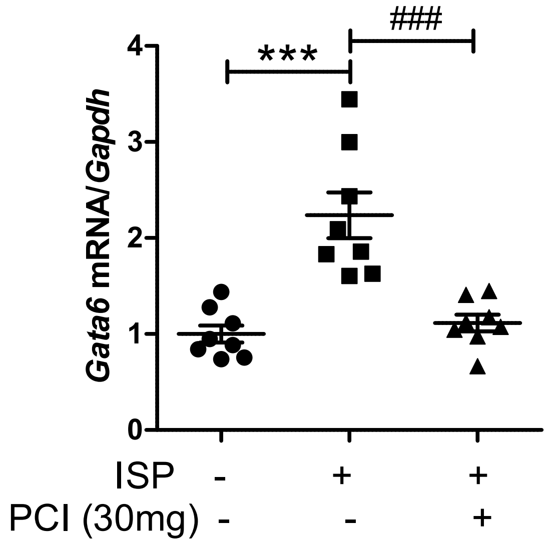

Supplementary Figure 3

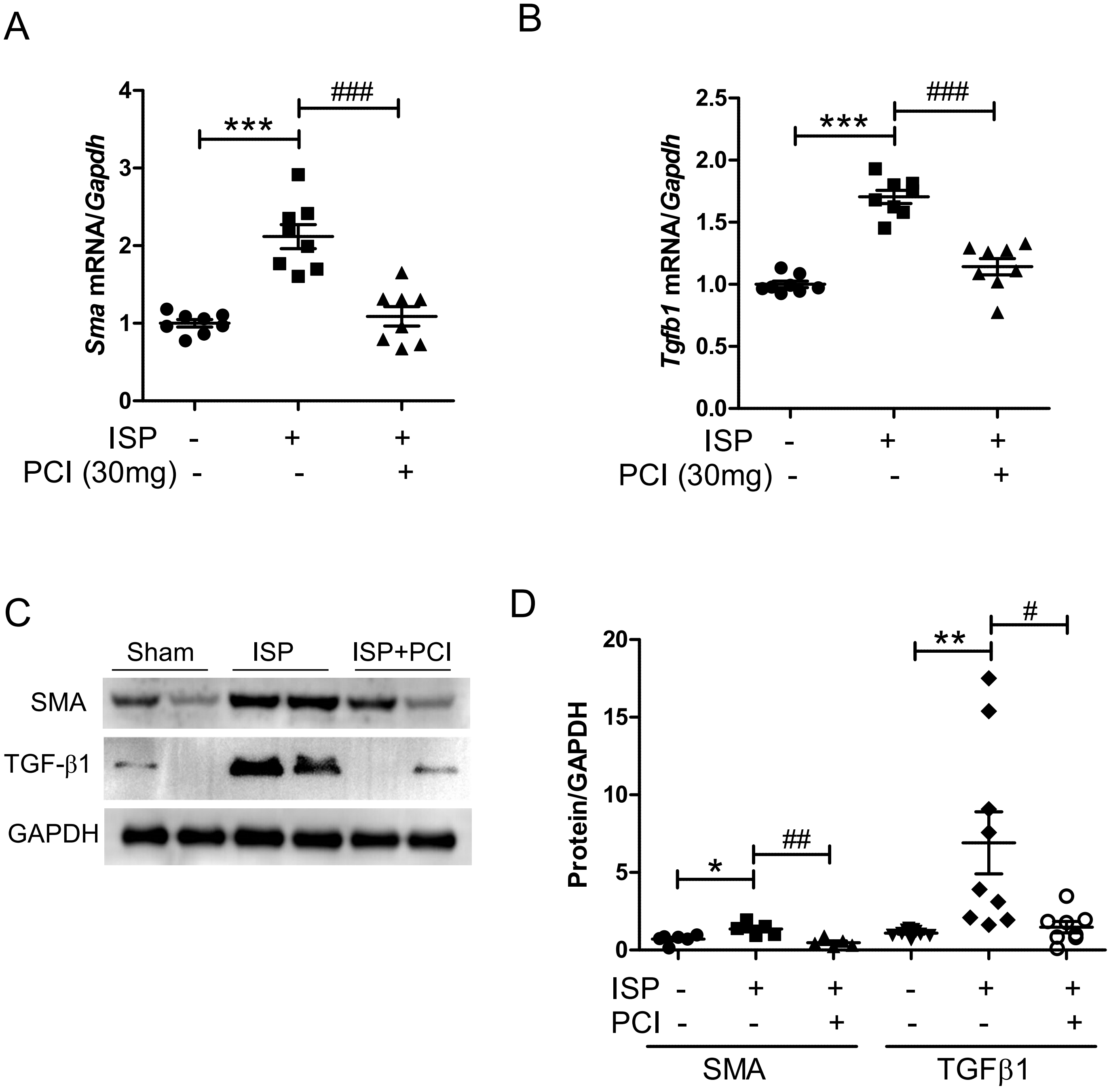

Supplementary Figure 4

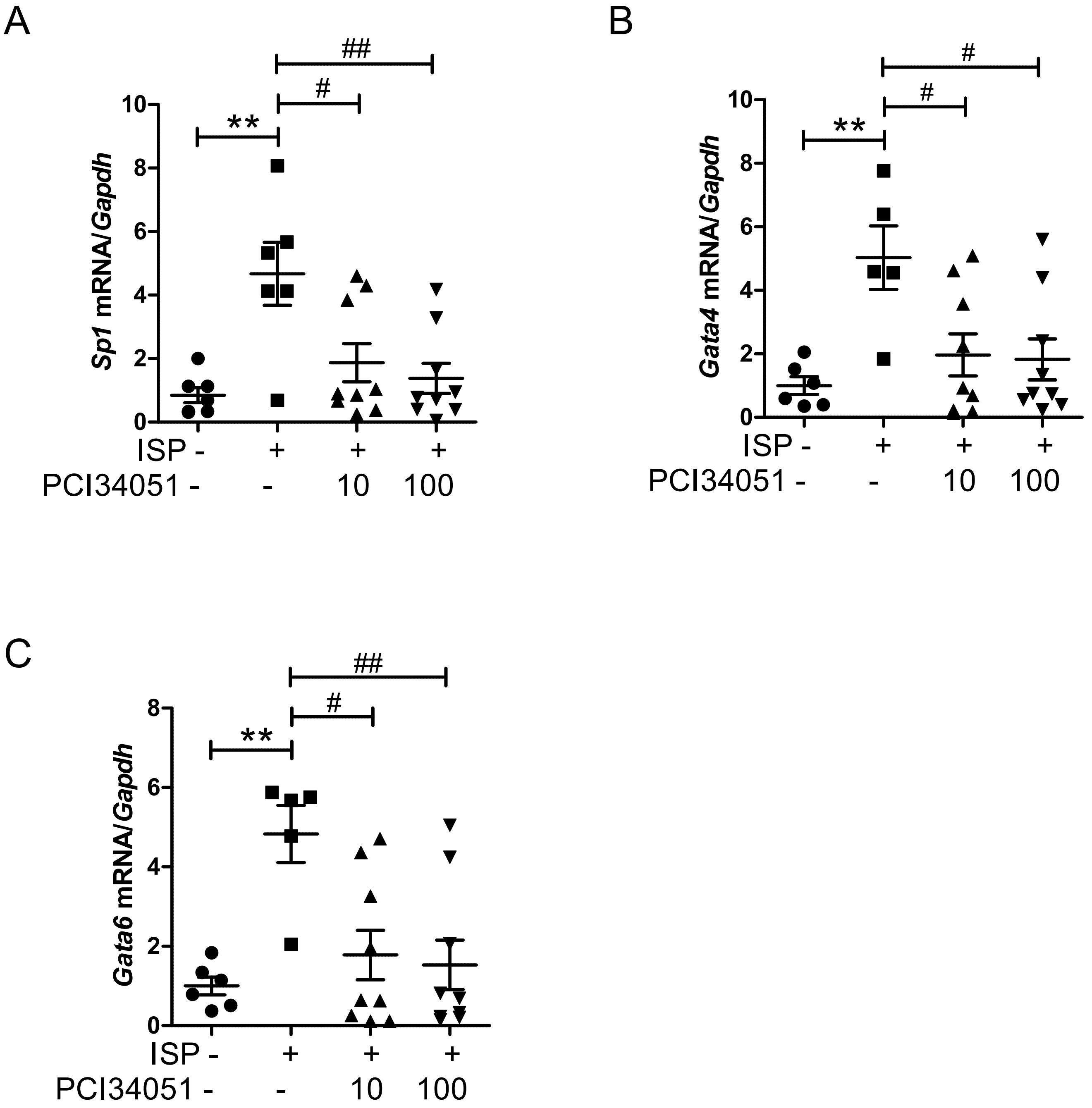

Supplementary Figure 5

A

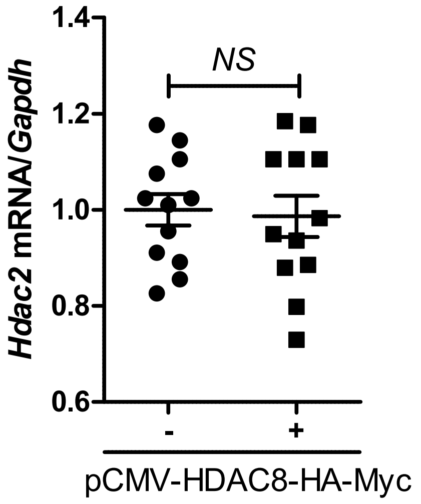

B

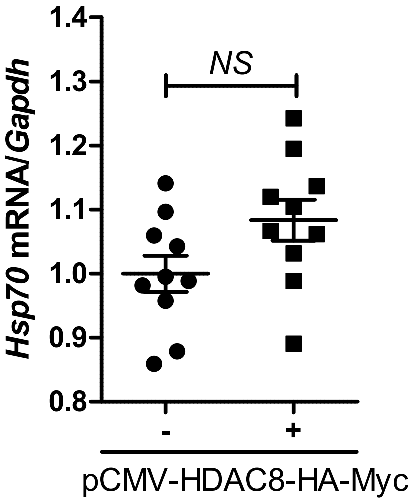

Supplementary Figure 6

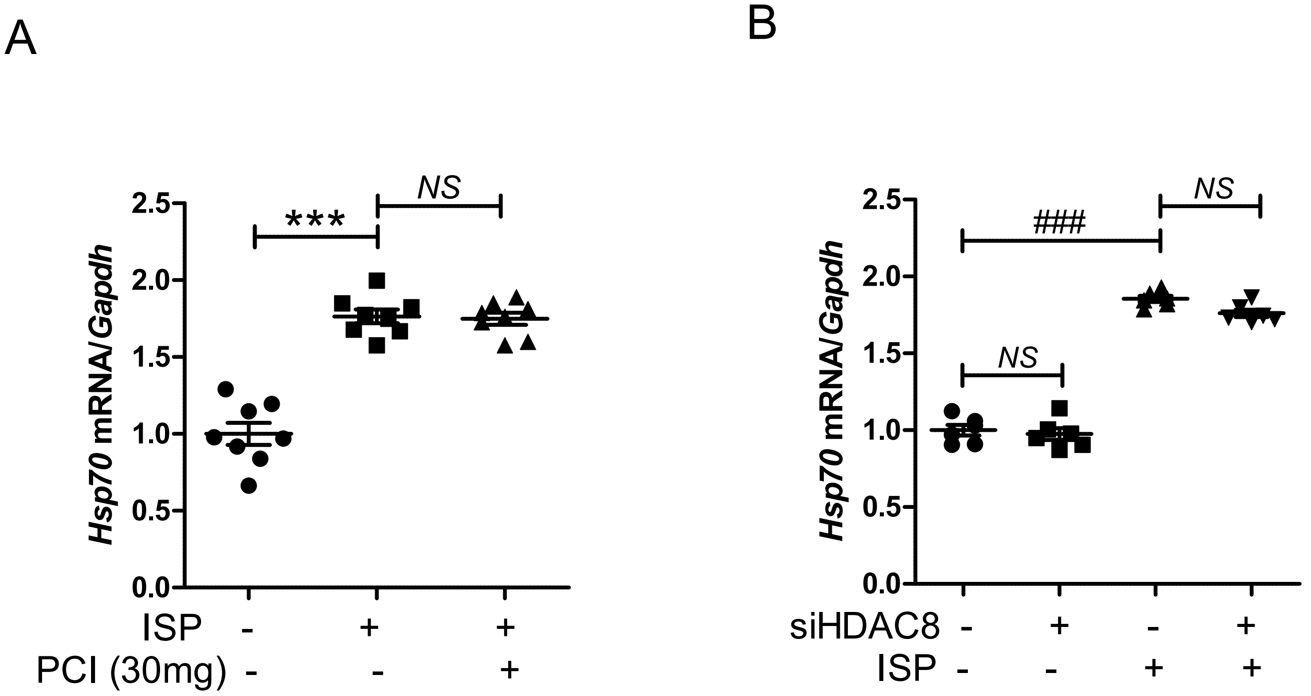

Supplementary Figure 7

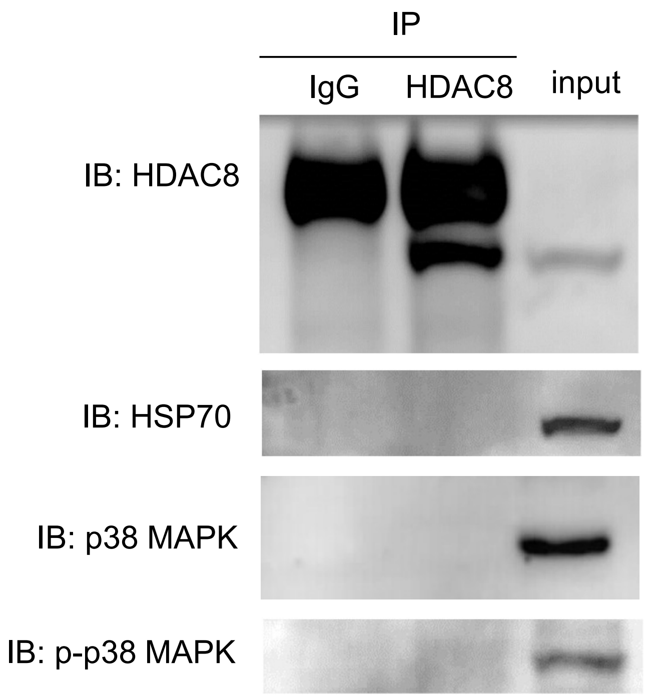

Table 1. Primers for RT-PCR

Gene	Primer sequence (5′ to 3′)
Gapdh (rat)	F: AACCCATCACCATCTTCCAGGAGC<br>R: ATGGACTGTGGTCATGAGCCCTTC
Nppa (rat)	F: GCTCGAGCAGATCGCAAAAG<br>R: GAGTGGGAGAGGTAAGGCCT
Nppb (rat)	F: GACGGGCTGAGGTTGTTTTA<br>R: ACTGTGGCAAGTTTGTGCTG
Myh7 (rat)	F: CCTCGCAATATCAAGGGAAA<br>R: TACAGGTGCATCAGCTCCAG
Sp1 (rat=mouse)	F: TCTGCAGCTACCCTGACTCC<br>R: TAATTCCCATGTTGCTGGTG
Gata4 (rat)	F: AGTCCTGCACAGCCTACCTG<br>R: GCCGGTTGATACCATTCATC
Gata6 (rat)	F: CTACACTTCCCATCCCTTCG<br>R: CGAGCGTCTGGTACATTTCC
Hsp70 (rat)	F: ACCAACCACCTCAAGCAAAG<br>R: GTCATTCCGTTCCTTCTCCA
Hdac2 (rat)	F: CTGCACCACGCCAAGAAGTCAGA<br>R: CAGTTAGGTTGAAGCAGCCCAGCC
Collagen I (mouse)	F: GAGCGGAGAGTACTGGATCG<br>R: GCTTCTTTTCCTTGGGGTTC
Fibronectin (mouse)	F: GATGCACCGATTGTCAACAG<br>R: TGATCAGCATGGACCACTTC
Ctgf (mouse)	F: CAAAGCAGCTGCAAATACCA<br>R: GGCCAAATGTGTCTTCCAGT

Hdac8 (mouse)	F: TCCGAAGGCAGTGGTTTTAC<br>R: GATGACCCCGGTCAAGTATG
Gapdh (mouse)	F: GCATGGCCTTCCGTGTTCCT<br>R: CCCTGTTGCTGTAGCCGTATTCAT
Nppa (mouse)	F: TGGAGGAGAAGATGCCGGTAGAAGAT<br>R: AGCGAGCAGAGCCCTCAGTTTGCT
Nppb (mouse)	F: CTGAAGGTGCTGTCCCAGAT<br>R: GTTCTTTTGTGAGGCCTTGG
Myh7 (mouse)	F: GCATTCTCCTGCTGTTTCCT<br>R: CCCAAATGCAGCCATCTC
Gata4 (mouse)	F: CTGTGCCAACTGCCAGACTA<br>R: ATTCAGGTTCTTGGGCTTCC
Gata6 (mouse)	F: GCCAACTGTCACACCACAAC<br>R: GTTACCGGAGCAAGCTTTTG
Sma (mouse)	F: ACTGGGACGACATGGAAAAG<br>R: AGAGGCATAGAGGGACAGCA
Tgfb1 (mouse)	F: CTCTCCACCTGCAAGACCAT<br>R: ACGCGGGTGACCTCTTTAG
